# Hydrolipidic Characteristics and Clinical Efficacy of a Dermocosmetic Formulation for the Improvement of Homeostasis on Oily Mature Skin

**DOI:** 10.3390/life13010087

**Published:** 2022-12-28

**Authors:** Letícia Kakuda, Maísa Oliveira de Melo, Patrícia Maria Berardo Gonçalves Maia Campos

**Affiliations:** School of Pharmaceutical Sciences of Ribeirão Preto, University of São Paulo, Ribeirão Preto 14040-903, SP, Brazil

**Keywords:** skin homeostasis, oily skin, biophysical techniques, clinical study, imaging analysis cosmetic formulation

## Abstract

Background: Although the scientific literature associates mature skin with dry skin and the secretion of sebum on the face decreases over the years, in tropical countries, such as Brazil, mature skin can still present oily characteristics. Thus, the knowledge of the hydrophilic characteristics of mature skin is fundamental to help the development of more effective treatments for this skin type. In this context, the study aimed to evaluate the hydrophilic characteristics and the clinical efficacy of a cosmetic formulation for mature skin added with alfalfa and lentil extracts by using biophysical and skin imaging techniques. Methods: Twenty-eight healthy females aged between 45 and 59 years were enrolled. Measurements of the stratum corneum water content, sebum content, transepidermal water loss, skin microrelief, and pores count were performed before and after the 28-day formulation application. Results: The mature skin presented as oily with wrinkles and pores. The proposed formulation significantly reduced the sebum content and the number of fine and large pores and improved skin microrelief and hydration after a 28-day period of the application when compared to the vehicle. Conclusions: The proposed formulation was effective in oily mature skin treatment, improving its general skin aging and oiliness conditions, and reducing pores count in just 28 days.

## 1. Introduction

Skin aging is a natural and gradual process that involves external and internal factors and their interactions, also known as exposome [1,2]. Among the exposome factors, solar radiation is well established as an aggressive element that accelerates skin aging [1]. Direct exposure to UV radiation increases the reactive oxygen species (ROS) in the skin, promoting skin photodamage [3]. In general, aged skin is known to present with characteristics such as the loss of elasticity, increased wrinkles, progressive dryness, and alterations in the skin’s hydrophilic balance [4,5]. Additionally, the mature skin demonstrated a decrease in the cellular renewal of collagen, elastin, keratinocytes, and fibroblasts, compromising the structural integrity [6] and causing pore deformation [7].

Although the scientific literature associates mature skin with dry skin, the secretion of sebum on the face decreases over the years [4,5,8]. In tropical countries, such as Brazil, mature skin can still present oily characteristics due to the high temperatures promoted by the tropical climate [2,9,10]. This characteristic includes a shiny appearance, greasy skin, increased thickness, enlarged pores, and a higher tendency towards acne due to the overproduction of sebum caused by 5-α-reductase type 1 [7,9,10]. In addition, the shiny appearance and dilated pores are evident on the face, negatively affecting self-esteem and compromising the quality of life [9,11]. Additionally, it can be found in the literature that an alteration in sebum production promotes a disbalance in the hydrolipidic mantle, leading to changes in cutaneous homeostasis [2].

This way, knowing that oily skin is the most remarkable skin type in Brazil [9], oily mature skin needs cosmetics products that are developed according to their specific conditions, with clinically proven efficacy that involve not only the improvement of general aging characteristics but also the oiliness aspects [2].

Consumers nowadays are advocating for the incorporation of natural bioactives, such as natural extracts or functional ingredients, which promote several benefits in a single product [12,13]. In this context, it is evident how knowing the needs of a specific skin type directs the choice of active ingredients that can act in the prevention and treatment of these conditions, as is the case of oily mature skin.

Therefore, the use of natural ingredients such as alfalfa extract (*Medicago sativa*) and lentil extract (*Lens esculenta*), which due to their composition of galactomannans and oligosaccharides, present potential to be applied in the development of innovative dermocosmetic formulations for oily mature skin [14].

The lentil extract (*Lens esculenta*) is rich in oligosaccharides and in phenolics compounds, such as epicatechin glucoside, tannins, procyanidin dimers, quercetin diglycoside, and trans-p-coumaric acid [15,16], which are described in the literature as substances that can reduce skin oiliness and attenuate the appearance of dilated pores by restoring the keratinization process and limiting the deformation and loosening of the skin [15,16]. On the other hand, the alfalfa extract contains isoflavones in its composition, which can act as a potent antioxidant agent [17] and has the potential to stimulate cellular activity and promote epidermal renewal by regulating the differentiation of keratinocytes that is lost during the aging process [15,18]. Formononetin, genistein, irilone, tricin, diadzein, Biochanin A, 5′-methoxysativan, coumarin derivatives (coumestrol, medicagol, sativol, trifoliol, lucernol, daphnoretin), and pectin methylesterase are the most abundant isoflavones present in the alfalfa extract (*Medicago sativa*) [19,20,21].

Furthermore, biophysical and skin imaging techniques have been applied for skin characterization and to evaluate the clinical efficacy of dermocosmetic products once they allow skin analysis in vivo, in real-time, and in a non-invasive way [2,22]. Thus, these techniques are essential tools in evaluating the benefits of dermocosmetic treatments for oily, mature skin.

In this context, the aim of the study was to evaluate the clinical efficacy of a dermocosmetic formulation containing a combination of alfalfa and lentil extracts in the improvement of hydration, wrinkles, and pores on oily mature skin by using biophysical and skin imaging techniques.

Finally, considering that there are very few products for mature skin that also consider the oiliness characteristics, this study presents an important contribution to the knowledge and development of effective dermocosmetic products for mature skin with the persistence of a high number of apparent pores.

## 2. Materials and Methods

### 2.1. Studied Formulations

A gel cream formulation based on Cetearyl Alcohol and Dicetyl Phosphate and Ceteth-10 Phosphate, Acrylates/C10–30 Alkyl Acrylate Crosspolymer, Cyclomethicone (and) Dimethicone Crosspolymer, Cyclopentasiloxane, C12–15 Alkyl Benzoate, Glycerin, Propypeleneglicol, aqua, BHT, EDTA, and Phenoxyethanol was developed. This formulation was added or not (vehicle—F1 to 1% of alfalfa (*Medicago sativa*) with 1% of the lentil (*Lens esculenta*) extracts in combination (F2) (Silab, France), as described in Table 1.

### 2.2. Study Design

The study was conducted according to the guidelines of the Declaration of Helsinki and approved by the Ethics Committee of the School of Pharmaceutical Sciences (CEP/FCFRP nº. 411—CAAE nº 56378216.0.0000.5403). All of the participants were informed and instructed about the objectives and methods of the investigation, and informed consent was obtained from all of the subjects involved in the study.

Twenty-eight healthy female participants aged 45 and 59 (mean ± SD: 51.82 ± 4.92) were enrolled in the study. The inclusion criteria were given as healthy Caucasian females with oily skin aged between 45–59 with Fitzpatrick phototypes between II and III. The exclusion criteria were smoking, pregnancy or lactation, use of drugs that can produce an abnormal skin response, and localized or generalized dermatological diseases.

Before the beginning of the study, 44 subjects were evaluated to confirm if they had oily skin. To detect and quantify the amount of sebum on the face, the Sebumeter^®^ SM815 photometer (Courage & Khazaka, Köln, Germany) was used in the frontal and malar areas [23]. Values above 150 µg/cm² for each region were considered oily skin [2]. Overall, 28 participants were selected, and instrumental measurements were performed in terms of the stratum corneum water content, sebum content, and transepidermal water loss on the malar and frontal regions of the face.

After that, the study participants were divided into two groups, which were randomized with 14 participants each. The first one received the formulation added to the extracts under study (F2), and the other group received the vehicle formulation (F1). The participants applied the formulation once a day at night before washing their faces. Measurements in terms of the stratum corneum water content, sebum content, transepidermal water loss were performed on the malar and frontal regions of the face before and after 28 days of formulations application. In addition, pore count and skin microrelief were evaluated on the malar region, and a wrinkles score was performed on the periorbinal region of the face.

The study was a randomized double-blind, placebo control, and it was performed on the frontal and malar regions of the face on the right or left side. The measurements were taken immediately after 20 min of acclimatization in a room with controlled temperature and humidity (temperature 22 ± 2 °C and humidity 45 ± 2%) [9].

### 2.3. Clinical Study—Instrumental Measurements

The stratum corneum water content was determined by a Corneometer^®^ CM 825 (Courage & Khazaka, Germany) using the water capacitance principle [2,9]. The results were given in arbitrary units (A.U.). The transepidermal water loss (TEWL) measurements were taken using a Tewameter^®^ TM210 device (Courage & Khazaka, Germany). This equipment is considered to be important to the analysis of the epidermal barrier function that measures the TEWL, which measures the gradient of water evaporation on the skin surface [24]. The results are presented in g/h/m².

Visioscan^®^ VC 98 (Courage & Khazaka, Germany) is a high-resolution UVA-light video camera that is used to evaluate skin microrelief. This equipment provides qualitative and quantitative information relating to the skin surface under physiological conditions, using optical profilometry techniques through image scanning. With this method, it is possible to evaluate the parameters related to the surface of the skin (SELS—Surface Evaluation of Living Skin), such as skin smoothness (SEsm), scaliness (Sesc), and wrinkles (SEw) [24,25].

The sebum content was evaluated in the frontal and malar regions of the participants using Sebumeter^®^ SM810 (Courage & Khazaka, Germany) equipment. For this purpose, an opaque tape was pressed into the skin for 30 s with slight pressure to collect the sebum. The region with the sebum makes the tape transparent. This area is calculated, and the value represents the sebum amount on the skin’s surface [9].

**Figure 1:**
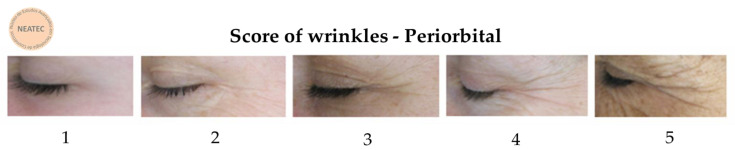
Periorbital wrinkles score developed by the NEATEC group.

**Figure 2:**
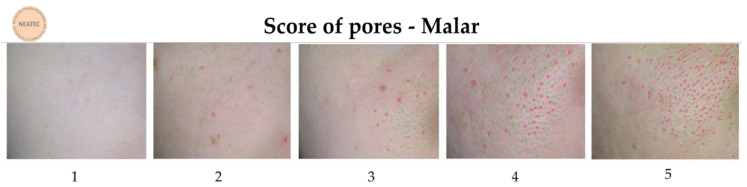
Pore score (count fine in green and count large in red) developed by the NEATEC group.

The Visioface^®^ RD (Courage & Khazaka, Germany) device was used to analyze the skin surface’s visible alterations through a high-resolution image of the face illuminated by white LEDs and light-emitting diodes (UV-like LEDs) [9]. To obtain the images, the participants had their faces placed in 3 defined positions on the device (front, right side, and left side), and the camera was fixed in front of the face to avoid the influence of different angles or distances during the analysis [26]. With these images, the VisioFace^®^ RD software calculates the number of fine and large pores in the malar region [9] and obtains 3D images of the periorbital area to analyze according to a score developed by our research group (NEATEC—Center of Advanced Studies in Cosmetic Technology). The score is composed of 5 points (1 to 5) and was used to classify the appearance of the wrinkles (Figure 1). In addition, a score of pores, also from our research group, was used to classify the pore quantity (Figure 2), with the fine count being: 1: 0–8, 2: 9–53, 3: 54–235, 4: 236–419, and 5: >420 pores; for the large count: 1: 0–5, 2: 6–10, 3: 11–85, 4: 86–152, 5: >153. The results are reported as the relative frequency (percentage) [9].

### 2.4. Statistical Analysis

The statistical analysis was performed using GraphPad Prism 8.4.3 (San Diego, CA, USA). The Shapiro–Wilk test was used to evaluate the data distribution (normality test). Normal distribution: one-way ANOVA with Tukey post-test was used. Non-normal distribution: Kruskal–Wallis with Dunn’s post-test was used. A *p*-value < 0.05 was considered significant.

## 3. Results

In the screening carried out before the beginning of the study, no variation was noticed between the analyzed regions. This means that the analyzed group of oily mature skin presents a homogeneity in the sebum content, the stratum corneum water content, and the transepidermal water loss values on different areas of the face, is considered oily. Thus, it is possible to compare the results obtained in the clinical study using the proposed formulation and to evaluate the effects in both regions (Table 2).

### 3.1. Stratum Corneum Water Content

**Figure 3:**
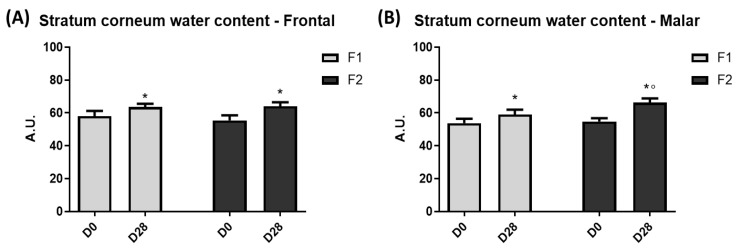
Stratum corneum water content at frontal (**A**) and malar (**B**) region before (D0) and after 28 days (D28) of application of F1 and F2. * Significant difference from baseline (D0) values (*p* < 0.05). ° Significant difference between F1 and F2 at D28 (*p* < 0.05).

In the malar and frontal areas, the stratum corneum water content presented with a significant (*p* < 0.05) improvement for both groups after 28 days (D28) in relation to their baseline values (D0). However, when comparing the groups at D28, the F2 showed a significant increase in this parameter in the malar region (Figure 3).

### 3.2. Transepidermal Water Loss

No statistical difference (*p* > 0.05) was noticed in the transepidermal water loss after 28 days of the study for both groups and regions (Table 2), presenting mean values of 11.20 and 10.88 at D28 for the frontal and malar areas, respectively.

### 3.3. Skin Microrelief Analysis

**Figure 4:**
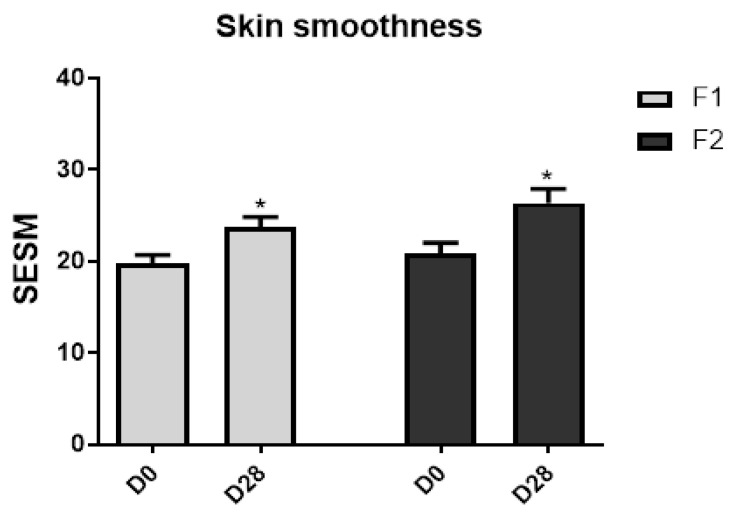
Skin smoothness before (D0) and after 28 days (D28) of application of F1 and F2 in the malar region. * Significant difference from baseline (D0) values (*p* < 0.05).

**Figure 5:**
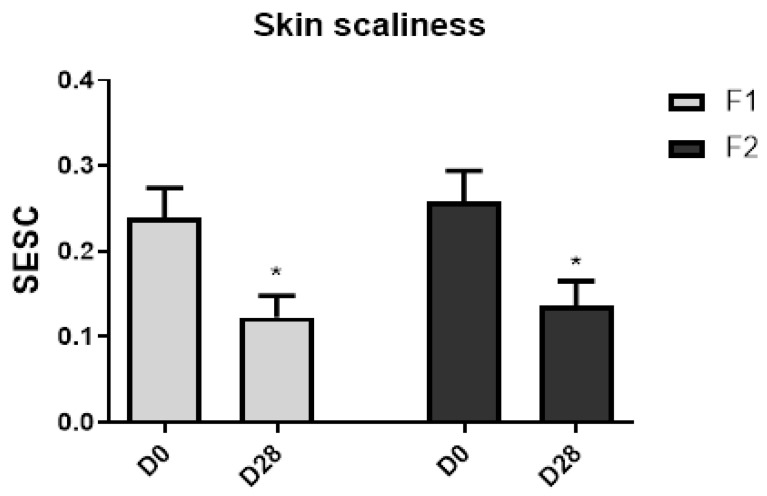
Skin scaliness before (D0) and after 28 days (D28) of application of F1 and F2 in the malar region. * Significant difference from baseline (D0) values (*p* < 0.05).

The skin smoothness parameter and skin scaliness showed a significant improvement (*p* < 0.05) in the malar and frontal regions after 28 days of study for both groups (Figure 4 and Figure 5).

**Figure 6:**
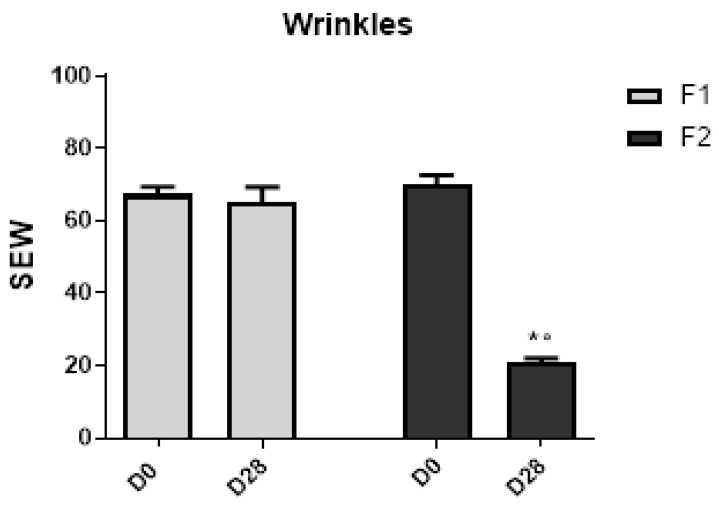
Skin microrelief SEw parameter—number and width of the wrinkles before (D0) and after 28 days (D28) of application of F1 and F2 in the malar region. * Significant difference from baseline (D0) values (*p* < 0.05). ° Significant difference between F1 and F2 at D28 (*p* < 0.05).

The number and width of the wrinkles significantly (*p* < 0.05) improved only for F2 in relation to their baseline value. When comparing the groups at D28, a significant (*p* < 0.05) difference was also noted (Figure 6) in the malar area.

### 3.4. Sebum Content

**Figure 7:**
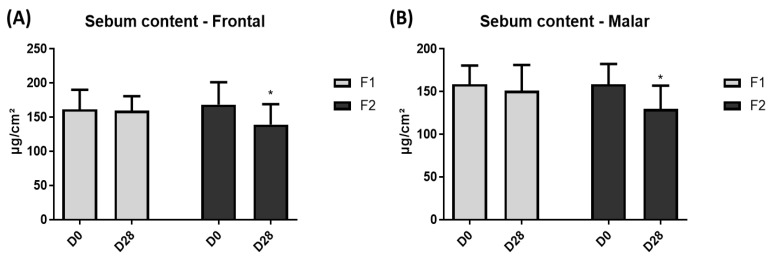
Sebum content at frontal (**A**) and malar (**B**) region before (D0) and after 28 days (D28) of application of F1 and F2. * Significant difference from baseline (D0) values (*p* < 0.05).

A significant difference in the sebum content was observed in the malar and frontal regions only for F2 (Figure 7).

### 3.5. Pores Amount and Pore Score

**Figure 8:**
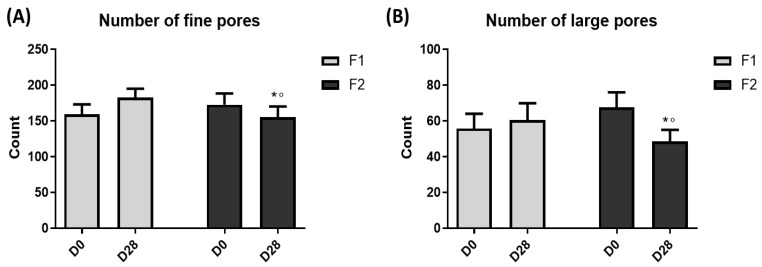
Number of fine (**A**) and large (**B**) pores before (D0) and after 28 days (D28) of application of F1 and F2. * Significant difference from baseline (D0) values: *p* < 0.05. ° Significant difference between F1 and F2 at D28 (*p* < 0.05).

**Figure 9:**
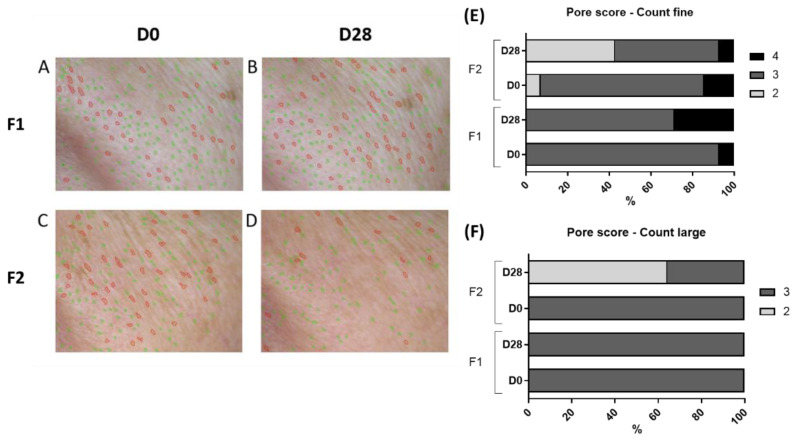
Representative images of the number of fine (green circles) and large (red circles) pores before (D0) and after 28 days (D28) of application of F1 (images (**A**,**B**)) and F2 (images (**C**,**D**)); Pore score: Count Fine (**E**) and Count Large (**F**).

The number of fine and large pores reduced significantly (*p* < 0.05) at D28 only for F2 (Figure 8 and Figure 9). Additionally, for the F2 formulation, the score for the pores reduces from 3.07 to 2.64 for fine pores and from 3.07 to 2.36 for large pores.

### 3.6. Score of Wrinkles

**Figure 10:**
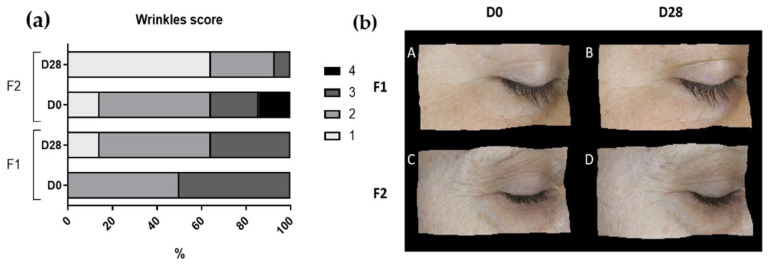
Periorbital wrinkles score (**a**) and representative images of the wrinkles (**b**) before (D0) and after 28 days (D28) of application of F1 (images (**A**,**B**)) and F2 (images (**C**,**D**)).

The participants of both groups had an average of 2.4 scores for the appearance of periorbital wrinkles at D0. After 28 days of formulation application, the score for the group that used the F1 formulation was 2.2, and the group using the F2 formulation showed an improvement in the wrinkles, decreasing the score to 1.7 (Figure 10).

## 4. Discussion

A previous study from our research group, Melo & Maia Campos (2018) [2], applied biophysical and skin imaging techniques to characterize oily mature skin. The authors concluded that oily skin presents different characteristics from normal/dry skin in terms of sebum content, microrelief parameters, and dermis thickness and that the future products developed specifically for this skin type should aim at these parameters. The 28-period of the study was determined to evaluate if the antioxidant properties of the combination of both extracts can improve cell turnover [27].

In this context, we proposed using the association of the lentil and alfalfa extracts (natural active substances) in a dermocsometic formulation due to their benefits for oily and aged skin, respectively. Considering that the choice of raw materials directly influences the consumer’s acceptance [28], we proposed the use of a gel cream formulation, as it is a well-accepted vehicle for people with mature skin [14]. The vehicle formulation was designed to not influence the skin barrier function or the maintenance of hydrolipidic balance [29,30].

The improvement in the stratum corneum water content can be related to the ingredients of vehicle formulation, such as glycerin, which has a humectant property and can hydrate and restore the function of the stratum corneum [31,32]. However, when comparing the groups at time D28, the group that used the F2 formulation showed a significant increase in this parameter compared to F1. This pronounced hydration effect can be associated with the composition of the extracts, such as oligosaccharides, which can also promote skin hydration [14,25,29,33]. In addition, the TEWL did not present significant differences for both formulations, which suggests the maintenance of the skin barrier function integrity during the use of the developed formulations [2].

In terms of skin microrelief, the skin smoothness parameter (SEsm) represents a relationship between the width and form of the wrinkles, and with cosmetic treatments, especially for aged skin, an increase in this parameter is expected, improving the texture and smoothness of the skin [34]. The increase in this parameter for both formulations can be explained due the presence of lipids, silicones, polymers, and other ingredients in the vehicle formulation, which are known to act in the moisturizing process and the improvement of skin microrelief [35,36,37].

The skin scaliness parameter (Sesc) shows the level of dryness of the stratum corneum and its flaking [34]. Both formulations under study showed a statistically significant reduction in the Sesc parameter. This suggests that a synergic effect between the vehicle and the active substances helped to reduce the skin scaliness in the malar region under the study conditions. This result corroborates with the increase in the SEsm, as the Sesc evaluation presents with reduced skin scaliness, which is related to skin smoothness and associated with the hydration parameters [24,34].

However, in the SEw parameter—the number and width of the wrinkles—which is calculated from the proportion of horizontal and vertical wrinkles [13], only the F2 showed a significant decrease in this parameter when compared to baseline values and F1 after the 28 days of study. It is described in the literature that cosmetic formulations with oligosaccharides may have a moisturizing effect, which can improve skin microrelief [25].

These results corroborate the improvement of the appearance of wrinkles, showing a beneficial effect on the skin’s microrelief [14].

Furthermore, the aging process leads to a loss of skin elasticity due to reduced collagen fibers, which could result in an increased pore count [38]. This way, the observed decrease in pores count can be related to improved skin aging, and products with this characteristic should be considered for oily, mature skin.

The alfalfa extract has a rich composition in isoflavone, presenting an antioxidant effect in topical formulations [15]. This property can be related to the acceleration of cell renewal [6,39]. The isoflavone could interrupt the free radical chain reactions and improve cell renewal [6]. Additionally, this extract stimulates collagen synthesis contributing to reducing wrinkles [24,40,41,42].

The presence of the lentil extracts in the F2 formulation could reduce the sebum content and the number of fine and large pores due to its composition in tannis and epicatechins [15]. These secondary metabolites are known for their astringent and antioxidant properties, respectively [9,15,16]. The tannin acts on sebum control by suppressing the production of surface lipids and the secretion of sebum [11]. Additionally, tannin has been reported to be effective in inhibiting 5-α-reductase enzymes in vitro [7]. This enzyme is responsible for the overproduction of sebum on the skin and promotes enlarged pores [7]. This way, the presence of tannins in the lentil extract can act on the sebum control and reduce the pore count.

Additionally, the reduced pores can be related to the properties of the lentils and the alfalfa extracts that act in restoring the synthesis of collagen, benefiting skin elasticity and appearance, as this improved elasticity can be associated with the number of fine and large pores of the skin [7,13,25]. This is an important result once enlarged pores are a feature that is the target of many complaints, as it interferes with the cosmetic application, creating a non-homogeneous surface.

In addition, the maintenance and balance of the hydrolipidic mantle are related to the control of oiliness since the excessive increase or decrease compromises this mantle, unbalancing the barrier function [43]. Thus, the association of the extracts improved oil control, skin microrelief, and a significant reduction in pore count, ensuring hydrolipidic balance and maintaining skin homeostasis.

Finally, improving the general conditions of mature skin is not enough for the population living in tropical countries, which may experience an increase in sebum production, leading to an increase in the number of pores on the face. These characteristics are directly related to the health of the skin, as there is an imbalance in the hydrolipidic mantle. In addition, dilated pores and the greasy and shiny appearance of the face generate social discomfort and affect self-esteem, especially for women. Thus, the use of natural ingredients, such as the extracts under study, can not only act on the general condition of mature skin but also the improve oiliness and dilated pores that can be persistent in adulthood.

## 5. Conclusions

The participants showed a high amount of superficial sebum, which leads to hydrolipidic disruption and an imbalance in skin homeostasis. Thus, a specific formulation was developed to attend to oily mature skin needs, containing natural ingredients. The formulation F2 containing the lentil and alfalfa extracts, in combination, improved the skin microrelief for reducing the Sew parameter. In addition, the proposed formulation reduced the number of fine and large pores after a 28-day period of study. In this context, the lentil and alfalfa extract can be suggested as effective ingredients for mature skin with overproduction of sebum and enlarged pores. The obtained results after only four weeks of use are important to customer adhesion to the use. In addition, biophysical and skin imaging techniques were effective tools to evaluate the skin physiology parameters related to hydrolipid balance and clinical efficacy of studied formulations using instrumental measurements in a non-invasive way and under real-time conditions.

## Figures and Tables

**Table 1:** Studied formulations.

I.N.C.I. ¹ Name	F1	F2
	%	%
Cetearyl Alcohol and Dicetyl Phosphate and Ceteth-10 Phosphate	5	5
Acrylates/C10–30 Alkyl Acrylate Crosspolymer	0.2	0.2
Cyclomethicone (and) Dimethicone Crosspolymer	5	5
Cyclopentasiloxane	15	15
Phenoxyethanol	0.6	0.6
Disodium EDTA	0.05	0.05
Butil Hidroxi Tolueno	0.01	0.01
Propypeleneglicol	4	4
Glycerin	3	3
C12–15 Alkyl Benzoate	5	5
Water & *Lens esculenta* (Lentil) Seed Extract	-	1
Water & *Medicago sativa* (Alfalfa) Extract	-	1
Distilled water	qs 100	qs 100

¹ International Nomenclature of Cosmetic Ingredient.

**Table 2:** Characterization of the participants in terms of sebum content, stratum corneum water content and transepidermal water loss in the frontal and malar region (Mean ± SD).

Measurement	Frontal	Malar	*p* Value
Sebum content (µg/cm²)	164.75 ± 30.48	158.37 ± 22.55	0.3776
Stratum corneum water content (A.U.)	56.78 ± 11.78	54.30 ± 8.53	0.3699
Transepidermal water loss (g.m^−2^.h^−1^)	11.26 ± 2.79	11.44 ± 3.41	0.8337

## Data Availability

The data can be shared upon request.

## References

[1] Krutmann J., Bouloc A., Sore G., Bernard B.A., Passeron T. (2017). The skin aging exposome. J. Dermatol. Sci..

[2] Melo M.O., Maia Campos P.M.B.G. (2018). Characterization of oily mature skin by biophysical and skin imaging techniques. Skin Res. Technol..

[3] Chen J., Liu Y., Zhao Z., Qiu J. (2021). Oxidative stress in the skin: Impact and related protection. Int. J. Cosmet. Sci..

[4] Vergilio M.M., Vasques L.I., Leonardi G.R. (2021). Characterization of skin aging through high-frequency ultrasound imaging as a technique for evaluating the effectiveness of anti-aging products and procedures: A review. Skin Res. Technol..

[5] Dulal S.R., Taher M.A., Sheikh H. (2019). Sandalwood Oil Can Be a Miraculous Tackle on Skin Aging, Skin Appearance and Wrinkle Skin-A Review. World J. Pharm. Med. Res..

[6] Shirata M.M.F., Maia Campos P.M.B.G. (2021). Sunscreens and Cosmetic Formulations Containing Ascorbyl Tetraisopalmitate and Rice Peptides for the Improvement of Skin Photoaging: A Double-blind, Randomized Placebo-controlled Clinical Study. Photochem. Photobiol..

[7] Son D.H., Nam M.H., Hong C.O., Seol H.M., Yang J.E., Kim Y.B., Kim C.T., Lee K.W. (2013). 5-α reductase inhibitory effect and astringent activity of green apple rind extract on human keratinocytes and fibroblast cells. Biosci. Biotechnol. Biochem..

[8] Tončić R.J., Kezić S., Hadžavdić S.L., Marinović B. (2018). Skin barrier and dry skin in the mature patient. Clin. Dermatol..

[9] Gabarra M.A.L., Maia Campos P.M.B.G. (2020). Correlations between sebaceous glands activity and porphyrins in the oily skin and hair and immediate effects of dermocosmetic formulations. J. Cosmet. Dermatol..

[10] Sakuma T.H., Maibach H.I. (2012). Oily skin: An overview. Skin Pharmacol. Physiol..

[11] Pongsakornpaisan P., Lourith N., Kanlayavattanakul M. (2019). Anti-sebum efficacy of guava toner: A split-face, randomized, single-blind placebo-controlled study. J. Cosmet. Dermatol..

[12] Ahmed I.A., Mikail M.A., Zamakshshari N., Abdullah A.S.H. (2020). Natural anti-aging skincare: Role Potential. Biogerontology.

[13] Gianeti M.D., Maia Campos P.M.B.G. (2014). Efficacy evaluation of a multifunctional cosmetic formulation: The benefits of a combination of active antioxidant substances. Molecules.

[14] Shirata M.M.F., Maia Campos P.M.B.G. (2016). Importance of texture and sensorial profile in cosmetic formulations development. Surg. Cosmet. Dermatol..

[15] Dorni A.C., Amalraj A., Gopi S., Varma K., Anjana S.N. (2017). Novel cosmeceuticals from plants—An industry guided review. J. Appl. Res. Med. Aromat. Plants.

[16] Amarowicz R., Estrella I., Hernández T., Robredo S., Troszyńska A., Kosińska A., Pegg R.B. (2010). Free radical-scavenging capacity, antioxidant activity, and phenolic composition of green lentil (*Lens culinaris*). Food Chem..

[17] Jing C.L., Dong X.F., Tong J.M. (2015). Optimization of ultrasonic-assisted extraction of flavonoid compounds and antioxidants from alfalfa using response surface method. Molecules.

[18] Campos P.M.M., Benevenuto C.G., Calixto L.S., Melo M.O., Pereira K.C., Gaspar L.R. (2019). *Spirulina*, *Palmaria Palmata*, *Cichorium Intybus*, and *Medicago Sativa* extracts in cosmetic formulations: An integrated approach of in vitro toxicity and in vivo acceptability studies. Cutan. Ocul. Toxicol..

[19] Aktaş T., Çölgeçen H., Havva A.T.A.R. (2021). Formononetin Production by Large-Scale Cell Suspension Cultures of *Medicago sativa* L.. Int. J. Second Metab..

[20] Barreira J.C., Visnevschi-Necrasov T., Nunes E., Cunha S.C., Pereira G., Oliveira M.B.P. (2015). *Medicago* spp. as potential sources of bioactive isoflavones: Characterization according to phylogenetic and phenologic factors. Phytochemistry.

[21] Nabatchian F., Aghoosi S.M.H., Mordadi A., Khodaverdi F. (2015). Evaluation of the effect of alfalfa extract on breast cancer. J. Appl. Environ. Biol. Sci..

[22] Verschoore M., Nielson M. (2017). The Rationale of Anti-Aging Cosmetic Ingredients. J. Drugs Dermatol..

[23] Ahn K., Han S., Yun K., Lee W., Lee D.-G., Kang S.M., Choi Y.-B., Han K., Ahn Y.J. (2022). A Real-Time Detection Device for the Rapid Quantification of Skin Casual Sebum Using the Oil Red O Staining Method. Sensors.

[24] Maia Campos P.M.B.G., Mercurio D.G., Melo M.O., Closs-Gonthier B. (2017). *Cichorium intybus* root extract: A “vitamin D-like” active ingredient to improve skin barrier function. J. Dermatolog. Treat..

[25] Shirata M.M.F., Maia Campos P.M.B.G. (2017). Influence of UV filters on the texture profile and efficacy of a cosmetic formulation. Int. J. Cosmet. Sci..

[26] Hernandez C.A., Espinal J.M., Zapata D.U., Coimbra D., Alfertshofer M., Frank K., Green J.B., Davidovic K., Gavril D.L., Cotofana S. (2021). The Influence of Different Light Angles During Standardized Patient Photographic Assessment on the Aesthetic Perception of the Face. Aesth. Plast Surg..

[27] Martini A.M., Maia Campos P.M.B.G. (2018). Influence of visible light on cutaneous hyperchromias: Clinical efficacy of broad-spectrum sunscreens. Photodermatol. Photoimmunol. Photomed..

[28] Calixto L.S., Maia Campos P.M.B.G. (2017). Physical–Mechanical characterization of cosmetic formulations and correlation between instrumental measurements and sensorial properties. Int. J. Cosmet. Sci..

[29] Coltelli M.B., Danti S., De Clerck K., Lazzeri A., Morganti P. (2020). Pullulan for advanced sustainable body-and skin-contact applications. J. Funct. Biomater..

[30] Mahrhauser D., Nagelreiter C., Baierl A., Skipiol J., Valenta C. (2015). Influence of a multiple emulsion, liposomes and a microemulsion gel on sebum, skin hydration and TEWL. Int. J. Cosmet. Sci..

[31] Mokrejs P., Hutta M., Pavlackova J., Egner P., Benicek L. (2017). The cosmetic and dermatological potential of keratin hydrolysate. J. Cosmet. Dermatol..

[32] Stettler H., Kurka P., Lunau N., Manger C., Böhling A., Bielfeldt S., Wilhelm K.P., Dähnhardt-Pfeiffer S., Dähnhardt D., Brill F.H. (2017). A new topical panthenol-containing emollient: Results from two randomized controlled studies assessing its skin moisturization and barrier restoration potential, and the effect on skin microflora. J. Dermatolog. Treat..

[33] Maia Campos P.M.B.G., Melo M.O., Junior F.B.C., Ramawat K.G., Mérillon J.M. (2015). Effects of Polysaccharide-Based Formulations on Human Skin. Polysaccharides.

[34] Enright K.M., Nikolis A. (2020). In vivo determination of the skin surface topography and biophysical properties of human hands: Effects of sex and hand dominance. Skin Res. Technol..

[35] Jeong C.B., Han J.Y., Cho J.C., Suh K.D., Nam G.W. (2013). Analysis of electrical property changes of skin by oil-in-water emulsion components. Int. J. Cosmet. Sci..

[36] Estanqueiro M., Conceição J., Amaral M.H., Lobo J.M., Grumezescu A.M. (2016). The role of liposomes and lipid nanoparticles in the skin hydration. Nanobiomaterials in Galenic Formulations and Cosmetics.

[37] Sundaram M.D.H. (2016). Pilot comparative study of the topical action of a novel, crosslinked resilient hyaluronic acid on skin hydration and barrier function in a dynamic, three-dimensional human explant model. J. Drugs Dermatol..

[38] Maia Campos P.M.B.G., Melo M.O., Calixto L.S., Fossa M.M. (2015). An oral supplementation based on hydrolyzed collagen and vitamins improves skin elasticity and dermis echogenicity: A clinical placebo-controlled study. Clin. Pharmacol. Biopharm..

[39] De Lima Cherubim D.J., Buzanello Martins C.V., Oliveira Fariña L., da Silva de Lucca R.A. (2020). Polyphenols as natural antioxidants in cosmetics applications. J. Cosmet. Dermatol..

[40] Rana M.G., Katbamna R.V., Padhya A.A., Dudhrejiya A.D., Jivani N.P., Sheth N.R. In vitro antioxidant and free radical scavenging studies of alcoholic extract of Medicago Sativa L. Rom. J. Biol.-Plant Biol. 2010, 55, 15–22.

[41] Silva L.R., Pereira M.J., Azevedo J., Gonçalves R.F., Valentão P., de Pinho P.G., Andrade P.B. (2013). Glycine max (L.) Merr., *Vigna radiata* L. and *Medicago sativa* L. sprouts: A natural source of bioactive compounds. Food Res. Int..

[42] Panchenko L., Muratova A., Turkovskaya O. (2017). Comparison of the phytoremediation potentials *of Medicago falcata* L. and *Medicago sativa* L. in aged oil-sludge-contaminated soil. Environ. Sci. Pollut. Res..

[43] Delsin S.D., Mercurio D.G., Fossa M.M., Maia Campos P.M.B.G. (2015). Clinical efficacy of dermocosmetic formulations containing Spirulina extract on young and mature skin: Effects on the skin hydrolipidic barrier and structural properties. Clin. Pharmacol. Biopharm..

